# Anaphylaxis Mimicking Severe Croup in Pediatric Patients: A Case Series

**DOI:** 10.7759/cureus.104431

**Published:** 2026-02-28

**Authors:** Hrithik Dakssesh Putta Nagarajan, Roshan P K, Md Ramij Biswas, Tejashvi Rameshkumar, Mirza Adnan Baig, Nitish Thirugnanasambandam, Naveen Sundar Rameshkumar

**Affiliations:** 1 Department of Internal Medicine, Madurai Medical College, Madurai, IND; 2 Department of Emergency Medicine, Government Medical College, Thiruvananthapuram, Thiruvananthapuram, IND; 3 Department of Internal Medicine, Rajshree Medical Research Institute, Bareilly, IND; 4 Department of Internal Medicine, K.A.P. Viswanatham Government Medical College, Tiruchirappalli, IND; 5 Department of Internal Medicine, Dr. RK Diabetic Foot and Podiatry Institute, Chennai, IND; 6 Department of Internal Medicine, Indiana University School of Medicine, Goshen, USA

**Keywords:** allergy and anaphylaxis, case report, croup, sea food allergy, spasmodic cough

## Abstract

Acute stridor is frequently equated with croup in pediatric contexts; however, other conditions, such as anaphylaxis, may present with remarkably similar clinical and radiographic characteristics. This article presents two pediatric cases of acute stridor initially managed as croup but ultimately diagnosed and treated as anaphylaxis induced by seafood ingestion. Both patients experienced recovery following the administration of intramuscular adrenaline. These cases underscore the necessity for increased vigilance and comprehensive history-taking when evaluating pediatric acute stridor. Identifying allergic triggers and administering timely treatment can be lifesaving.

## Introduction

Stridor is characterized by a high-pitched sound resulting from turbulent airflow through a constricted airway [1]. In pediatric populations, it is most commonly attributed to croup, which typically presents with a barking cough, hoarseness, inspiratory stridor, and subglottic narrowing (steeple sign) and is often caused by viral pathogens such as parainfluenza virus [2,3]. However, noninfectious etiologies may closely mimic this presentation. Anaphylaxis, a life-threatening systemic hypersensitivity reaction diagnosed clinically using established criteria, such as the National Institute of Allergy and Infectious Diseases and the Food Allergy and Anaphylaxis Network (NIAID/FAAN) guidelines, can cause acute laryngeal or subglottic edema, leading to stridor and respiratory distress [4].

Pediatric airway emergencies require rapid assessment, as delayed recognition of uncommon but serious conditions may result in significant morbidity. While wheezing is the more typical respiratory manifestation of anaphylaxis, predominant upper airway obstruction with stridor is uncommon and may create diagnostic uncertainty. This case series highlights a rare presentation of seafood-induced anaphylaxis initially managed as severe croup and underscores the importance of maintaining a broad differential diagnosis in children presenting with apparent croup-like stridor.

## Case presentation

Case 1

**Figure 1:**
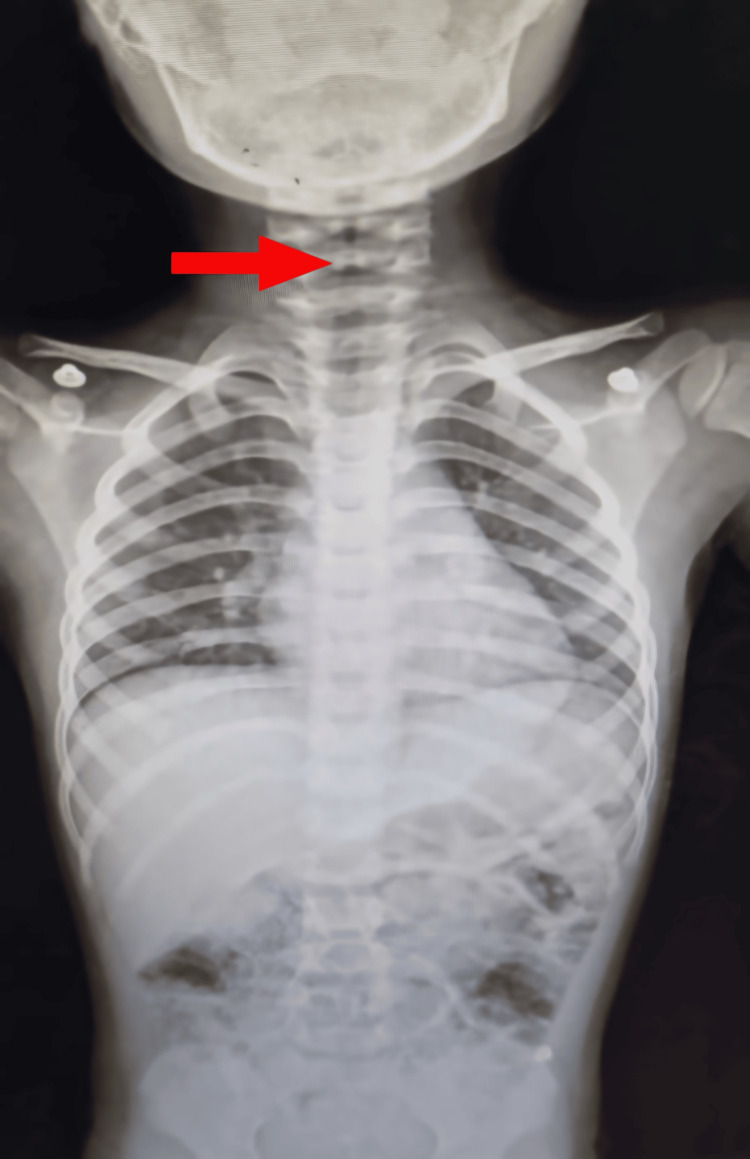
Posteroanterior chest radiograph demonstrating subglottic narrowing (steeple sign). The red arrow highlights the tapered narrowing of the upper trachea. This radiographic appearance mimics croup due to allergic subglottic edema.

A seven-year-old child presented to the emergency department with an acute onset of noisy breathing and dyspnea. Clinical examination revealed pronounced inspiratory stridor and a characteristic barking cough. The respiratory rate was 40 breaths/minute, with an oxygen saturation of 85% on room air. Additionally, a decrease in bilateral air entry was noted. The pulse rate was 140 beats/minute, and the blood pressure was 100/60 mmHg. The patient was afebrile, and no urticaria, angioedema, flushing, or rash was observed. Also, the patient had no prior history of asthma, atopy, or allergy. Symptoms developed within the first hour of first-time squid ingestion, with progressive respiratory distress prompting emergency department presentation. The chest radiograph revealed a steeple sign, and the Westley croup score was calculated to be eight, indicating severe croup (Figure 1) [5]. Nebulized adrenaline and intravenous dexamethasone (0.6 mg/kg) were administered shortly after arrival; however, stridor and respiratory distress persisted. Intramuscular adrenaline was subsequently administered approximately 30 minutes later due to ongoing airway compromise. Further history revealed a familial predisposition to allergies involving certain pharmaceuticals and seafood.

Case 2

A 10-year-old male presented with acute-onset stridor and a severe croup-like cough, accompanied by multiple episodes of vomiting and abdominal pain. His respiratory rate was 34 breaths/minute, oxygen saturation was 84% on room air, pulse was 145 beats/minute, and blood pressure was 100/70 mmHg. Respiratory symptoms began within hours of first-time oyster ingestion and progressed over approximately four hours prior to emergency department arrival. There were no indications of prior respiratory infection. No urticaria, angioedema, flushing, or rash was observed.

Neck imaging revealed a steeple sign similar to that observed in Case 1; radiographic findings were obtained from documented medical records due to archival data loss. Initial treatment with nebulized adrenaline and intravenous hydrocortisone (100 mg) resulted in minimal improvement, and intramuscular adrenaline was initiated approximately 40 minutes later due to refractory airway compromise.

Investigations and differential diagnoses

Laboratory investigations demonstrated normal hemoglobin levels (12.5 g/dL), white blood cell counts (8500 and 8600 cells/µL), and C-reactive protein levels (1.1 and 1.0 mg/dL). Serum IgE levels were elevated (130 IU/mL and 150 IU/mL), and allergy panels were positive for squid and oyster proteins, respectively (Table 1). Mild elevation of total IgE was considered nonspecific and not diagnostic of acute anaphylaxis; specific IgE testing was performed after clinical stabilization to identify likely allergen sensitization. Key laboratory and imaging findings for both cases are summarized in Table 1. Serum tryptase was not obtained during the acute episodes, representing a potential limitation of this report.

**Table 1:** Comparison of laboratory and imaging findings between the two cases. Laboratory values were obtained during initial emergency department evaluation. Specific IgE testing was performed after clinical stabilization to identify likely allergen sensitization. Reference ranges correspond to standard laboratory values and may vary between laboratories.

Parameter	Reference Range	Case 1	Case 2
Section A: Laboratory Parameters			
Hemoglobin (g/dL)	11.5-13.5	12.5	12.5
Total White Blood Cell Count (cells/µL)	5,000-10,000	8,500	8,600
Neutrophils (%)	40-60	57	50
Lymphocytes (%)	20-40	37	29
Eosinophils (%)	1-5	3	4
Platelet Count (×10⁹/L)	150-450	280	300
C-reactive Protein (mg/dL)	<3	1.1	1.0
Serum IgE (IU/mL)	<100	130	150
Specific IgE to Seafood	Negative	Positive to squid proteins	Positive to oyster proteins
Section B: Imaging Findings			
Chest and Neck Radiograph	-	Subglottic narrowing (steeple sign); no pneumonia	Subglottic narrowing (steeple sign); no pneumonia
Bedside Airway Ultrasound	-	Subglottic edema with turbulent airflow (Video 1)	Not performed

Bedside airway ultrasonography in Case 1 demonstrated subglottic edema with turbulent airflow (Video 1). Ultrasonography was not performed in Case 2, as radiographic findings and clinical data were considered conclusive.

**Video 1:** Bedside airway ultrasonography demonstrating subglottic edema with turbulent airflow in Case 1. The ultrasound (transverse view) of the airway reveals edematous vocal cords and turbulent airflow through the constricted subglottic region. This visual evidence supports allergic airway edema with turbulent airflow, distinguishing it from typical croup presentations.

Case 2 fulfilled NIAID/FAAN clinical criteria for anaphylaxis based on acute respiratory compromise with associated gastrointestinal symptoms following exposure to a likely allergen. Case 1 was classified as probable respiratory-predominant anaphylaxis based on acute airway compromise after seafood ingestion and rapid clinical improvement following intramuscular epinephrine. Although Case 1 does not strictly meet current NIAID/FAAN criteria, there is increasing recognition that anaphylaxis may present with isolated respiratory or cardiovascular symptoms, supporting this diagnostic interpretation [6].

Alternative causes of acute stridor, including viral croup, were considered less likely due to the absence of fever and normal inflammatory markers. Foreign body aspiration was unlikely given the lack of choking history. Bacterial tracheitis and epiglottitis were deemed improbable due to clinical stability, absence of toxic appearance, and rapid response to epinephrine.

Treatment

Case 1

Nebulized adrenaline and dexamethasone yielded minimal alleviation of stridor. Upon the likelihood of anaphylaxis, 0.3 mg of 1:1000 adrenaline was administered intramuscularly. Clinical improvement was observed within minutes, with normalization of oxygen saturation from 85% to 98% in room air and reduction in work of breathing. No additional doses of adrenaline were necessary.

Case 2

The patient was initially treated with nebulized adrenaline and intravenous hydrocortisone; however, respiratory distress and vomiting persisted. Consequently, three doses of intramuscular adrenaline (0.3 mg, 1:1000) were administered at 30-minute intervals, followed by a brief intravenous adrenaline infusion to address ongoing airway compromise. Blood pressure remained within age-appropriate ranges, and escalation of therapy was undertaken for refractory airway obstruction rather than hemodynamic instability. Progressive improvement in stridor and oxygenation occurred over the following hours. A single dose of ondansetron was administered to alleviate persistent nausea and vomiting. Clinical stability was achieved once adrenaline effectively reduced the airway edema.

Following clinical stabilization, both patients were discharged on the second hospital day with prescriptions for emergency adrenaline auto-injectors. Comprehensive counseling on dietary allergen avoidance was provided to minimize the risk of re-exposure to identified triggers. Their parents were instructed on the correct intramuscular administration of the auto-injectors into the anterolateral aspect of the mid-thigh in the event of future allergic reactions.

## Discussion

Stridor may be inspiratory, expiratory, or biphasic, depending on the anatomical level of airway obstruction: supraglottic involvement typically produces inspiratory stridor and is often associated with viral croup; lower tracheal obstruction results in expiratory stridor, and glottic or subglottic pathology leads to biphasic stridor [7,8].

Anaphylaxis can exhibit clinical features that closely resemble severe croup, particularly when upper airway edema predominates [9]. Although wheezing is more commonly associated with anaphylaxis, stridor may occur in severe presentations and reflects significant laryngeal or subglottic involvement and can mimic infectious upper airway obstruction [10,11]. Gastrointestinal symptoms, including vomiting and abdominal pain, coupled with a recent history of allergen exposure, can aid in distinguishing anaphylaxis from croup [12]. While Case 2 fulfilled NIAID/FAAN diagnostic criteria for anaphylaxis, Case 1 represented probable respiratory-predominant anaphylaxis. Emerging literature recognizes that anaphylaxis may present with isolated respiratory manifestations that do not strictly satisfy current diagnostic criteria, underscoring limitations of existing frameworks and the importance of clinical judgment in atypical presentations [6].

A retrospective study conducted by Patnaik et al. reported that foreign body aspiration was the leading cause of stridor in children, accounting for 38.8% of all cases. Additionally, among the 62 cases analyzed, only one instance of stridor was attributed to anaphylaxis. This observation supports the rarity of the cases discussed in our article [7].

The management of croup is contingent upon the severity of the condition, which can be evaluated using the Westley Croup Score. Treatment decisions are subsequently made based on this score. Standard therapeutic interventions for croup include the administration of nebulized racemic epinephrine and corticosteroids such as hydrocortisone or dexamethasone. In cases where impending respiratory failure is suspected, the patient may require intubation and mechanical ventilation [5,13].

In contrast, the treatment of anaphylaxis typically begins with the removal of the suspected trigger, if feasible, followed by the administration of parenteral fluids. Pharmacological management is then employed, utilizing agents such as adrenaline, prednisolone, dimetindene, and either salbutamol or terbutaline. Additionally, supplemental oxygen may be administered. In particularly severe instances, intubation and mechanical ventilation may become necessary. The therapeutic approach is determined by the severity of the symptoms and the specific characteristics of the patient [14].

In our cases, alternative causes of acute stridor had been excluded clinically, and persistence of symptoms despite standard croup therapy, together with recent seafood exposure and associated gastrointestinal symptoms, strengthened diagnostic reassessment toward anaphylaxis. Corticosteroids and nebulized adrenaline alone were insufficient, as mediator-driven airway edema requires immediate adrenergic support [15]. The rapid clinical improvement following intramuscular epinephrine underscores its critical role in reversing airway compromise and highlights key clinical clues for recognizing anaphylaxis in children presenting with apparent severe croup, including recent allergen exposure, poor response to croup therapy, associated gastrointestinal symptoms, absence of infectious features, and prompt response to epinephrine.

## Conclusions

These cases illustrate a potential diagnostic mimic of severe croup and underscore the importance of maintaining a high index of suspicion for anaphylaxis in pediatric patients presenting with acute stridor, particularly when symptoms are unresponsive to standard croup therapy or when there is a history of allergen exposure. Given the clinical overlap between anaphylaxis and croup, thorough history-taking, focused assessment of recent dietary or environmental triggers, and repeated clinical reassessment are essential. Prompt administration of intramuscular epinephrine is life-saving and should be considered when conventional treatment fails to produce the expected response. Although these findings cannot define prevalence or typical presentation, they emphasize the importance of recognizing atypical manifestations and maintaining a broad differential diagnosis to prevent delays in definitive care. Continued education and awareness among healthcare providers are vital to support early identification and effective management of such potentially life-threatening presentations.
